# Supplementary material for: Modeling the thermal behavior of photopolymers for in-space fabrication
Source: NPJ Microgravity. 2026 May 27;12:75. doi: 10.1038/s41526-026-00612-1 (PMC13493797; doi:10.1038/s41526-026-00612-1)
Supplement: Supplementary file 1 [file 41526_2026_612_MOESM1_ESM.pdf]

Supplementary Information

Modeling the Thermal Behavior of Photopolymers for In-Space Fabrication

Jonathan Ericson[a], Daniel Widerker[a], Eytan Stibbe[b], Mor Elgarisi[a], Yotam Katzman[a], Omer Luria[a], Khaled Gommed[a], Alexey Razin[a], Amos A. Hari[a], Israel Gabay[a,⸙], Valeri Frumkin[a,§], Hanan Abu Hamad[c], Ester Segal[c], Yaron Amouyal[d], Titus Szobody[e], Rachel Ticknor[f], Edward Balaban[f] & Moran Bercovici[a,g,*]

[a] Faculty of Mechanical Engineering, Technion - Israel Institute of Technology, Haifa, Israel

[b] The Rakia Mission, 3 Shadal St. Tel Aviv-Yafo, Israel

[c] Faculty of Biotechnology and Food Engineering, Technion - Israel Institute of Technology, Haifa, Israel

[d] Faculty of Materials Science and Engineering, Technion - Israel Institute of Technology, Haifa, Israel

[e] Department of Chemical and Biomolecular Engineering, Rice University, Houston, TX

[f] NASA Ames Research Center, Moffett Blvd., Moffett Field, CA, USA

[g] Department of Materials, ETH Zürich, Switzerland

[⸙] Current affiliation: School of Chemical and Biomolecular Engineering and School of Integrative Plant Science, Cornell University, Ithaca, NY, USA

[§] Current affiliation: Department of Mechanical Engineering, Boston University, Boston, MA, USA

[*] Corresponding author: moran.bercovici@mat.ethz.ch

S1. Supplementary Videos

All identifiable individuals appearing in the supplementary materials are co-authors or participants in documented research activities. Written informed consent for publication of images and videos was obtained where required.

Video1. **Summary of ISS Fluidic shaping experiment**

Video2. **Observations of photopolymer blistering and fume release in microgravity**

Video3. **Recreation of polymer blistering in the lab**

Video4. **Parabolic flight photopolymerization experiment**

We here report the material characterization measurements of: TJ-3704A, a commercial acrylate-based resin also sold under the brand name 'VidaRosa' (Dongguan Tianxingjian Electronic Technology Co., Ltd, Guandong, China), and NOA61 & NOA63 (Norland Optical Adhesive, Norland Products Incorporated, New Jersey, USA), which were used in this work.

S2. Thermal diffusivity, α, characterization by Laser Flash Analysis (LFA)

Laser flash analysis (LFA) is a widely used transient technique for measuring a material's thermal diffusivity and is considered one of the principal methods for thermal transport characterization in solids and liquids. In this method, a short laser pulse is applied to one face of a small (dia. ranging between ca. 6 to 25 mm), planar sample, and the resulting temperature rise is recorded on the opposite face as a function of time with an infra-red detector. By fitting the rear-face temperature response to an analytical heat-diffusion solution considering diverse conditions of heat loss (conduction through the samples' faces, radial edges, radiation, etc.) and pulse-time corrections, the through-thickness thermal diffusivity, α, can be extracted.[1]

In this work, we used LFA to characterize the thermal diffusivity of the photopolymer resins in both liquid and solid states at isothermal temperatures of 25 and 50 °C, by applying short (0.5 ms) Nd-YAG laser pulses. We implemented the measurements using a NETZSCH LFA-457 *MicroFlash* ® apparatus. We tested cylindrical polymer samples with a diameter of 12.7 mm, and a nominal thickness of 1.65 mm. Liquid polymer resin samples were measured with the use of dedicated aluminum sample containers (NETZSCH catalog number 6.256.4-91.6.00). Following typical procedure, we spray-coated all the samples front and rear faces with black carbon to ensure uniform laser absorption and emissivity and applied the Cape-Lehman method to correct for heat losses from both back and front faces of the samples as well as for the effects of finite pulse durations.[2] To assess measurement uncertainties and improve accuracy, we measured each sample N = 3 times at each temperature and material state. Our results are reported in Table S1. For the temperatures measured we found a negligible variation in the sample's thermal diffusivity. We found that the liquid polymer samples have a larger thermal diffusivity than their solid counterparts, however, as this variation is small, it leads to negligible effects on our thermal model simulation predictions. Thus, for simulations of photopolymer curing of these specific polymers, we treat the thermal diffusivity, α, as a material specific constant,

$$\alpha(\Phi, T) = \alpha_0, \qquad (S1)$$

where Φ is the material phase and T is its temperature. Future applications of our model to other photopolymer formulations should begin with similar material characterization. If such measurements reveal a strong variation in thermal diffusivity, we suggest expanding the modeled thermal diffusivity to a more general form by assuming a linear dependance on the material state Φ,

$$\alpha(\Phi, T) = \alpha(\Phi = 0, T) + \big(\alpha(\Phi = 1, T) - \alpha(\Phi = 0, T)\big)\Phi. \qquad (S2)$$

Table S1: Photopolymer thermal diffusivity (in $\mathrm{mm^2/s}$) as measured by LFA at 25 and 50 °C. We measured the polymers in both liquid and solid phase. Each sample was measured N = 3 times at each temperature and material state with the mean and standard deviation of these measurements reported below.

Photopolymer thermal diffusivity, α mm^2/s

	TJ-3704A				NOA61				NOA63			
	Solid		Liquid		Solid		Liquid		Solid		Liquid	
**Temperature ˚C**	mean	STD	mean	STD	mean	STD	mean	STD	mean	STD	mean	STD
**25**	0.086	0.003	0.145	0.055	0.097	0.004	0.163	0.049	0.078	0.014	0.224	0.006
**50**	0.073	0.006	0.023	0.084	0.084	0.004	0.14	0.022	0.073	0.013	0.202	0.009

S3. Specific heat capacity, c_p, characterization by Differential Scanning Calorimetry (DSC)

Differential Scanning Calorimetry (DSC) is a well-established technique for determining the specific heat capacity, c_p, of polymers and other materials as a function of temperature. In DSC, we simultaneously heat a sample and a reference material with a well-known heat capacity, while monitoring the heat flow in time into each one.[3]

In this study, we preformed DSC measurements (with a Mettler Toledo DSC 3) of our three photopolymer formulations in both liquid and solid phase, against a standard sapphire reference sample. The samples were heated from 25 to 100 °C, at a rate of 10 °C/min under a nitrogen atmosphere. Importantly, all liquid photopolymer resin samples were protected from light to prevent unintended photopolymerization during preparation and measurement.

As shown in Figure S1, we found that in both liquid and solid state, c_p values rise linearly with temperature. We also measured a significant decrease in c_p for TJ-3704A after solidification. In our model we assume linear dependence of the heat capacity on the material state, such that

$$c_p(\Phi, T) = c_p(\Phi = 0, T) + \left(c_p(\Phi = 1, T) - c_p(\Phi = 0, T)\right)\Phi\,, \tag{S3}$$

where the functions

$$c_p(0, T) = c_p(0, T = 0) + \frac{\partial c_p(0, T)}{\partial T} T \tag{S4a}$$

and

$$c_p(1, T) = c_p(1, T = 0) + \frac{\partial c_p(1, T)}{\partial T} T, \tag{S4b}$$

are defined by linear fits of the measured data as shown in the figure. The resulting parameter values for all characterized materials are listed in Table S2 below.

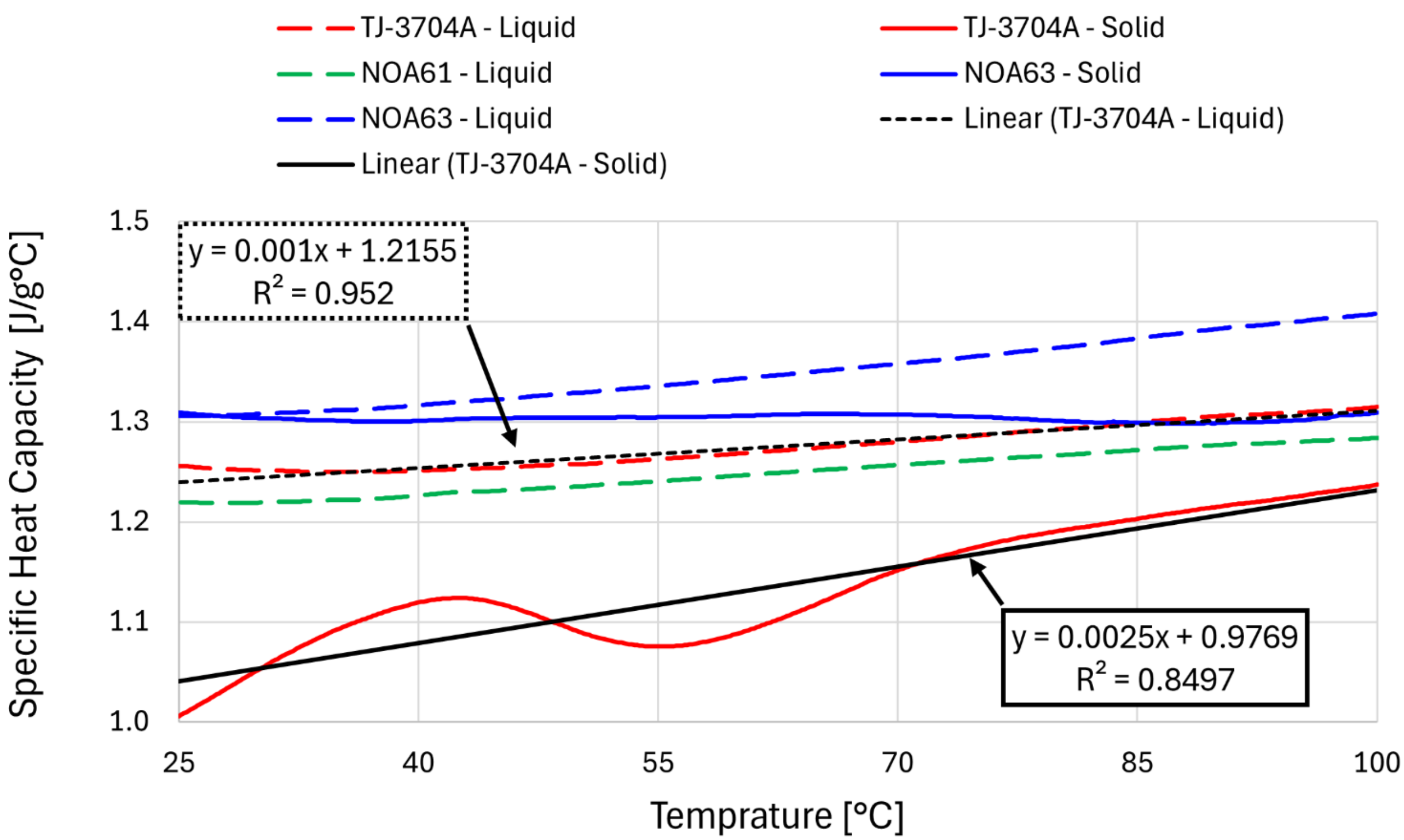

Figure S1: Polymer specific heat capacities as measured by DSC. We measured the polymer's specific heat capacity, c_p, in both liquid and solid phase from 25 to 100 °C at a rate of 10 °C/min, in a nitrogen atmosphere against a standard sapphire sample. The c_p dependance on temperature within the measured range was found to be linear. We measured a significant decrease in c_p for TJ-3704A (red) after solidification (solid red line in comparison to dashed red line). Based on these measurements we model the polymer's temperature dependent c_p for the liquid ($\Phi = 0$) and solid ($\Phi = 1$) phases as a linear function whose coefficients are prescribed by a fit of the measured data. The black lines in the plot present an example of such a fit for the liquid TJ-3704A (dashed black) and solid TJ-3704A (solid black). As shown in eq S3, by also assuming a linear dependence of the heat capacity on the material state we extend the use of these measurements to all intermediary polymerization states.

S4. Polymerization heat generation, $\dot{q}$, characterization by UV coupled Differential Scanning Calorimetry (UV-DSC)

Ultraviolet Differential Scanning Calorimetry (UV-DSC) enables quantitative measurement of the heat released during photopolymerization by combining conventional DSC heat-flow detection with in-situ UV irradiation. In this technique, the photopolymer resin is exposed to a controlled UV light intensity while the DSC records the exothermic heat flow associated with polymerization under a nitrogen atmosphere. By integrating the heat-flow signal over time, the total reaction enthalpy can be determined.[3]

In this work, we characterized the heat generation, $\dot{q}$, as a function of the polymerization state Φ and UV intensity I, for each of our polymers using a Mettler Toledo DSC-1 coupled with a Hamamatsu LC8-02 UV light source and a photocolorimetry kit. These measurements revealed typical non-monotonic autocatalytic behavior, where the reaction rate initially accelerates, reaches a peak, and then decelerates. We modeled this behavior with our autocatalytic model,

$$\dot{q} = \rho \Delta H \frac{\partial \Phi}{\partial t} = \dot{q}_0 \left(\frac{I}{I_{ref}}\right)^b \Phi^m (1-\Phi)^n, \qquad (S5)$$

where b, m, n and $\dot{q}_0$ are coefficients whose values we determine by data fitting, and I_{ref} is an arbitrary reference value for the intensity.

Figure S2 presents a surface plot of the autocatalytic model (heat release per unit mass as a function of polymerization state and light intensity) for TJ-3704A, overlayed by the measurements (black dots) that were used to determine its fitting parameters. The measured dependance on both UV intensity and polymerization is clearly well captured by our model. Furthermore, we note that for all three characterized polymers, such fitting resulted in typical R^2 values of 0.97 with root mean sum errors (RMSE) of less than 0.5 W/g. The resulting parameter values are listed in **Table S2** below.

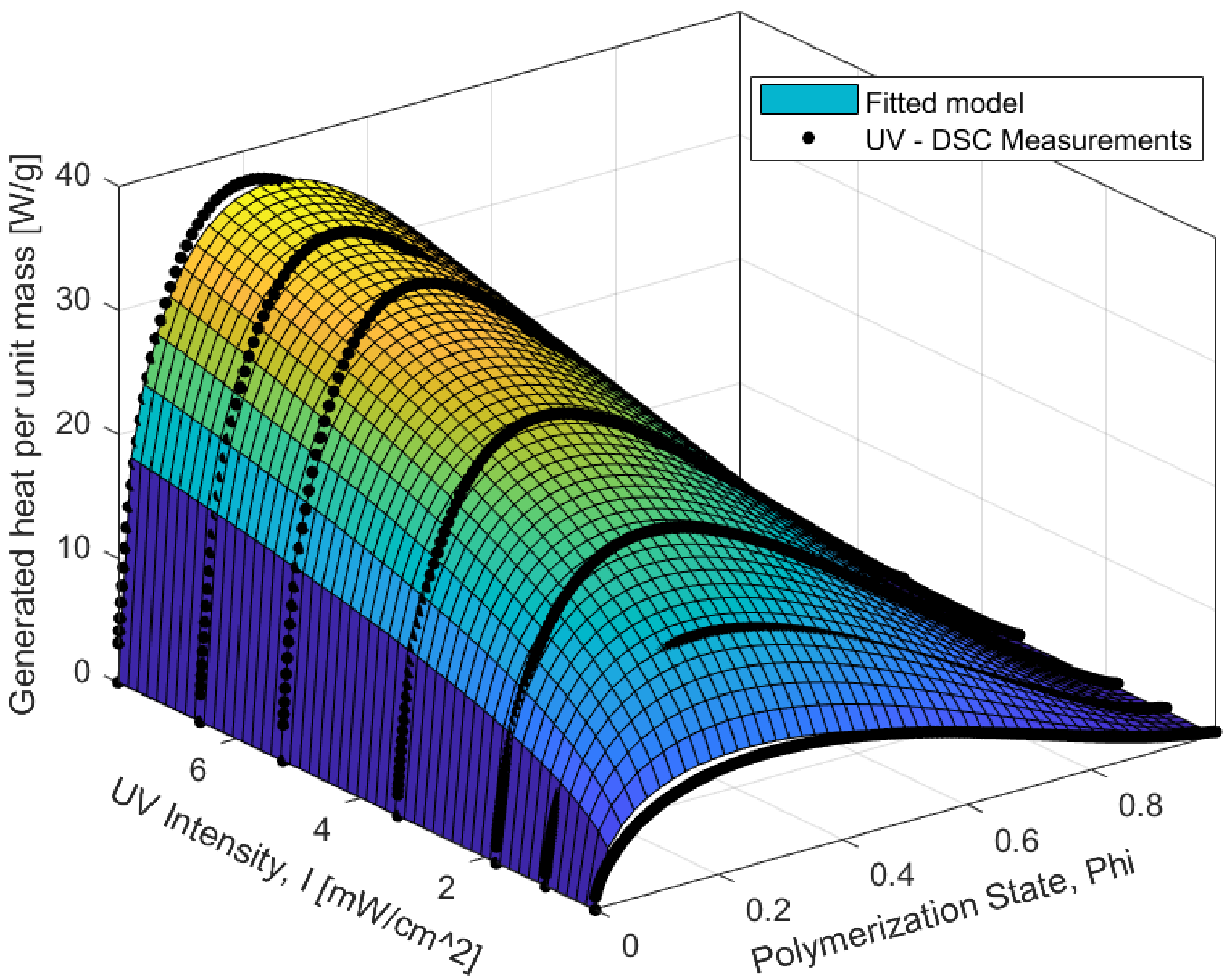

Figure S2: Heat generation model fitting to UV-DSC measurements (black dots) of TJ-3704A. The presented surface plot displays our model for the heat generation of TJ-3704A (normalized by its density, ρ) with parameters determined by fitting it to the measured data (black dots). We characterized all three photopolymers in the same manner. Fitting resulted in R^2 values of at least 0.97, with RMSEs lower than 0.5 W/g.

S5. Summary of material parameters

Table S2: Summary of material parameters

Property	Parameter	TJ-3704A	NOA61	NOA63	Units	Source
Density, ρ	ρ	1.079	1.260	~1.260	$\frac{g}{cm^3}$	Polymer MSDS
Specific heat capacity, c_p	$c_p(\Phi = 0, T = 0)$	0.9739	1.207	1.284	$\frac{J}{gK}$	DSC
	$c_p(\Phi = 1, T = 0)$	1.205	~1.207	1.292		
	$\frac{\partial c_p}{\partial T}(\Phi = 0)$	0.0025	0.0007	0.0011	$\frac{J}{gK^2}$	
	$\frac{\partial c_p}{\partial T}(\Phi = 1)$	0.0011	~0.0007	0.0003		
Thermal diffusivity, α	α_0	0.11	0.09	0.14	$\frac{mm^2}{s}$	LFA
Polymer heat release, $\dot{q}$	$\dot{q}_0$	57.82	69.53	90.43	$\frac{W}{cm^3}$	UV - DSC
	b	0.74	0.677	0.637	-	
	m	0.53	0.46	0.39	-	
	n	1.61	1.49	1.49	-	
Total polymer released heat, ΔH	H_∞	347	271	315	$\frac{J}{g}$	
	H_0	90	20	53		
	I_{sat}	13.77	38.25	38.53	$\frac{\mu W}{cm^2}$	
UV Absorption, β	$\beta(\Phi = 0)$	2.19	~2.19	~2.19	mm^{-1}	Transmission measurements
	$\beta(\Phi = 1)$	1.49	~1.49	~1.49		

~ Values which were assumed rather than directly measured

- Note: I_{ref} was set as $1\,\frac{mW}{cm^2}$.

S6. Chemical formulation of TJ-3704A, NOA61 and NOA63

Summarized below are the polymer resin chemical formulations as published by the manufacturers:

Table S3: TJ-3704A chemical formulation as published in MSDS by manufacturer

Element	CAS NO	Concentration %
Polyurethane acrylate prepolymer	9003-01-4	35 ~ 65
Acrylate monomers	7779-31-9	15 ~ 25
Acrylate monomers	27813-02-1	5 ~ 15
Photoinitiator A	7473-98-5	1 ~ 5
Photoinitiator B	947-19-3	1 ~ 2
Additives	68938-54-5	0.5 ~ 1

Table S4: NOA61 chemical formulation as published in MSDS by manufacturer

Element	CAS NO	Concentration %
Mercapto Ester	Trade Secret	50 ~ 65
Triallyl Isocyanuarte	1025-15-6	30 ~ 55

Table S5: NOA63 chemical formulation as published in MSDS by manufacturer

Element	CAS NO	Concentration %
Mercapto Ester	Trade Secret	50 ~ 75
Triallyl Isocyanuarte	1025-15-6	15 ~ 40

References

1. Blumm, J. The Laser Flash Technique: A Widespread Technology for Measurement of the Thermal Diffusivity of Solids and Liquids. *Int J Thermophys* **46**, 39 (2025).
2. Cape, J. A. & Lehman, G. W. Temperature and Finite Pulse-Time Effects in the Flash Method for Measuring Thermal Diffusivity. *J. Appl. Phys.* **34**, 1909–1913 (1963).
3. Höhne, G. W. H., Hemminger, W. F. & Flammersheim, H.-J. *Differential Scanning Calorimetry*. (Springer, Berlin, Heidelberg, 2003). doi:10.1007/978-3-662-06710-9.
